# Draft genome of clinical isolate of *bla*_OXA-395_ and *bla*_PAO_-producing *Pseudomonas aeruginosa* strain NeRAM02 isolated from urine in Niger

**DOI:** 10.1128/mra.01292-25

**Published:** 2026-03-16

**Authors:** Abdourahamane Yacouba, Oumou Hamidou, Ahmed Olowo-Okere, Abdoulaye Ousmane, Massir Issoufou-Noma, Bassirou Abdou-Natali, Ounoussa Tapha, Mahamadou Doutchi, Saidou Mamadou

**Affiliations:** 1 Faculty of Health Sciences, Abdou Moumouni University 486604 , Niamey, Niger; 2 National Reference Laboratory for Antimicrobial Resistance, Hôpital National Amirou Boubacar Diallo 667850 , Niamey, Niger; 3 Faculty of Pharmaceutical Sciences, University of Abuja 99399, Abuja, Federal Capital Territory, Nigeria; 4 Faculty of Health Sciences, Dan Dicko Dankoulodo University 665091, Maradi, Niger; 5 Faculty of Health Sciences, Andre Salifou University 553101 , Zinder, Niger; Loyola University Chicago, Chicago, Illinois, USA

**Keywords:** *Pseudomonas aeruginosa*, *bla*_OXA-395_, urine, draft genome, Niger

## Abstract

This study reports the draft genome of a clinical isolate of *Pseudomonas aeruginosa* NeRAM02, harboring *bla_OXA-395_* and *bla_PAO_*. The genome was 6.8 Mb with a GC content of 66.1%.

## ANNOUNCEMENT

Carbapenem-resistant *Pseudomonas aeruginosa* is classified as a “high-priority” pathogen by the World Health Organization (WHO) due to its global threat and limited treatment options (1). The emergence of *bla*_OXA-395_ and *bla*_PAO_ is particularly concerning due to their ability to confer resistance to last-resort antibiotics used to treat multidrug-resistant infections. Identification and surveillance of these high-risk clones are crucial for public health and the development of new antimicrobials. This study reports isolation of *Pseudomonas aeruginosa* harboring *bla*_OXA-395_ and *bla*_PAO_ in the Republic of Niger.

*P. aeruginosa* strain NeRAM02 was isolated from the clean-catch midstream urine specimen of an 85-year-old male patient in the Republic of Niger. The urine specimen was initially cultured on cystine–lactose–electrolyte-deficient (CLED) agar (Bio-Rad, Marnes-la-Coquette, France) and incubated aerobically at 37°C for 18–24 h. Distinct colonies were subsequently purified by repeated subculturing, and their susceptibility was determined using the Kirby-Bauer disc diffusion method as described by CLSI 2024 (2). Owing to its resistance to carbapenems and ceftazidime, the isolate was referred to the National Reference Laboratory for Antimicrobial Resistance for further characterization. Species identification was confirmed using the VITEK 2 compact (bioMérieux SA, Marcy-l’Étoile, France). The strain was thereafter sequenced at the Noguchi Memorial Institute for Medical Research (NMIMR), Accra, Ghana. Genomic DNA extraction, library preparation, and sequencing were performed as previously described (3). Briefly, genomic DNA of strain NeRAM02 was extracted using the QIAamp DNA extraction kit (Qiagen GmbH, Germany). DNA concentration was determined using the Qubit 1× dsDNA HS Assay on a Qubit 4 Fluorometer (Thermo Fisher Scientific, USA). Libraries were generated using the Illumina DNA Library Prep (M) Tagmentation Kit (Illumina Inc., San Diego, CA). Library quality and concentration were evaluated using the Agilent 2100 Bioanalyzer system and the KAPA Library Quantification qPCR Kit (Roche, California), respectively. All libraries were normalized to 2 ng/µL and subsequently pooled. The pooled libraries were sequenced on the Illumina NextSeq 1000 platform (Illumina Inc., San Diego, CA, USA) using 2 × 150 bp chemistry. Raw sequencing reads were processed using Trimmomatic v0.39 (4) to remove adapter sequences, trim low-quality bases (Phred score < 20), and discard reads shorter than 50 bp following quality filtering. The quality-filtered reads of 1,685,389 were then assembled *de novo* using SPAdes genome assembler v3.15.3 with default parameters (5). Genome completeness and contamination were determined using checkm2 v1.1.0 (6). The genome was annotated using the NCBI Prokaryotic Genome Annotation Pipeline v.6.10 (7).

Species identification was further validated by integrating results from FastANI v1.33 for pairwise average nucleotide identity (ANI) analysis, and EzAAI v1.2.3 for average amino acid identity (AAI) estimation, using the *P. aeruginosa* PAO1 (ASM676v1) genome as reference (8, 9). The PlasmidFinder v.2.1.6 was used to detect plasmid sequences in WGS (10). Subsequently, antibiotic resistance genes were identified using abricate v1.0.1 (https://github.com/tseemann/abricate) against the ResFinder version 2.0 (11). The results of the analysis are shown in Table 1. Ethical approval to conduct this study was granted by the National Ethics Committee for Health Research (CNERS), under the Ministry of Public Health, number 104/2024/CNERS.

**TABLE 1:** Assembly statistics and genomic metrics of *Pseudomonas aeruginosa* NeRAM02

Features	*P. aeruginosa* NeRAM02
Genome accession no.	JBRUJN000000000
BioSample accession no.	SAMN52836285
BioProject accession no.	PRJNA1346465
Average nucleotide identity (%)	98.7
Average amino acid identity (%)	100
Genome coverage (×)	16.0×
No. of reads	1,685,389
Completeness (%)	100
Contamination (%)	0.08
Genome size (bp)	6,772,385
Multilocus sequence typing	ST773
N50 (bp)	195,169
GC content (%)	66.1
Number of contigs	109
CDS	6,367
rRNA	3
repeat_region	2
tRNA	59
No. of genes	6,433
Predicted antimicrobial resistance genes	*cmlA9, aadA10 ,aph(3')-IIb ,bla_OXA-395_, bla_PAO_, catB7, fosA, qnrVC1, sul1, tet(G*)
Disinfectant genes	*qacE*

## Data Availability

Genome sequence reads have been deposited in the NCBI under the BioProject accession number PRJNA1346465. The Whole Genome Shotgun project has been deposited at DDBJ/ENA/GenBank under the accession number JBRUJN000000000. The version described in this paper is version JBRUJN000000000. Raw sequencing data are accessible in the SRA under the accession number SRX30984476.
